# 

**Published:** 1878-04

**Authors:** 


### INDEX.

### Vol. XIV to Vol. XVI inclusive.

### Contributions.

PAGE

Abuse of Stomach, Ben H. Pratt.. 1
Always Against the Women, Ausburn Towner .........64 and 120
A Man who did not Sweat, A. R. Killpatrick, M. D............ 67
As well as we Know, J. J. Keyes.. 146
About Livers, Ben H. Pratt...... 310
Because Others Do, Ben. H. Pratt. 115
Building the Language of Eternity, Ausburn Towner............ 257
Care of the Feet, P. H. Hayes, M.D. 62
Colds, P. H. Hayes, M. D......... 118
Child vs. Parent, Uncle Bob...... 122
Come What Say You? Thos. K. Beecher..................... 137
Crystals for the Microscope, Geo. E. Blackham, M. D., F. R. S. 259
Cheap Telescopes, Scientific American........................... 291
Diathesis, P. H. Hayes, M. D...... 5
Do the Fittest really Survive? Ausburn Towner................ 148
Exaggeration, J. B. Chandler,.... 260
How Shall We Train Our Boys? Uncle Bob........ ......... 38
Honor Bright, Ben. H. Pratt..... 87
Heels, Ben. H. Pratt........... 143
Honored Local Names, Ausburn Towner... .............. 229
Issue as a counter-irritant, P. H. Hayes, M. D................ 90
I Don't Know, Thos. K. Beecher.. 174
Laws Charlantanry, I, II, Ausburn Towner.....................284–313
Local Mic. Societies, Geo. E. Blackham, M. D., etc............. 317
Labor Saving, Thos. K. Beecher.. 316
My Doctor, J. J. Keyes.......... 180
My School Teacher, J. J. Keyes .. 231
Microscopic, I, II, S. O. Gleason, M. D......... ..........207-259
Monument to Adam, J. B. Chandler....... .................. 237
No Profession Exempt, Ausburn Towner...................... 92
New York Journalism, Ausburn Towner.... .... ... ... ..... 175
Old Age, Thos. K. Beecher....... 235
Piano Plaints, Ben. H. Pratt...... 60
Penology, J. B. Chandler......... 290
Reuben, Ausburn Towner........ 7
Success in Life, Ausburn Towner. 35
Specialties, J. J. Keyes.......... 205
Statistics, J. B. Chandler......... 208
Smile, A. J. J. Keyes............. 287
The Case of Brown, Ben. H. Pratt 31
The Game of Right Hand against Left, P. H. Hayes, M. D...... 34
Take of the Leaven of Damnation, Ben. H. Pratt............... 227
Ultraism in Music, Ben. H. Pratt. 172
Universality of Lying, Ben. H. Pratt......... ............. 283
What We Seem and What We Are. Ausburn Towner............ 202
Where shall we Recreate! Ben. H. Pratt..................... 254
Words, J. J. Keyes............... 316

### Medical Items and News.

Asthma, .........10–73–99–210–299–321
Acne.......................20–185–210
Arsenical Poisoning...... ........ 40
Arsenate of Gold ................ 42
Aromatic Syrup of Licorice....... 42
Antiseptic, local................. 44
" Balsam...........95–211
" Solution ........... 160
" dressing.............. 323
Anal Fissure........ ........... 70
Ascites.......................... 74
Antiasmatic Cigarettes........... 95
Alcoholism........ ..........98–102
Antiemetic....................... 125
Antispasmodic ...............126–210
Anginose Affections............. 126
Antipyretic .................... 127
Apomorphia ..................... 130
Anasarca....................... 158
Amorphia....................... 184
Antirheumatic Mixture........... 188
Alopecia and Psoriasis.......... 255
Anthelmintic, New........243-294-324
Amyl Nitrate.................... 297
Antiscorbutic Tincture.......... 239
Antineuralgic ................... 242
Boils..........................11–73
Bladder, injection of....... ..... 11
" Inflammation of........94–158
" Cleaning of............. 101
" Sedative for............. 153
" And Kidney Ailments.... 297
" Diseases of.............. 242
Blister.... ...... ..........12–159
Bright's Disease................. 13
Brain, needle in.................. 13
" experiments with.......... 215
Bay Rum, formula for........... 43
Bougies, soluable............... 43
Beer, sulphuric acid............. 44
Bromide Eruption............... 69
" of Ethyl.................. 155
Bichromate of Potash.. ......... 69
Bismuth Powder, compound...124–155
Batiator Root................... 133
Bronchitis.....................155–214
Benzoate of Lithia............... 156
Belladonna, as an intestinal remedy....... ......... ...... 183
Breast, Abscess of............... 211
Baldness......................... 216
Bile, in Urine................... 264
Burns..........................47–292
Bloodless Method of Operating... 246
Balsam Copaiba Emulsion.... ... 321
Cholera.......................10–98
Chloral, Danger of... .......... 10
Chloral, Syrup of................ 125
" Large Doses of.......... 41
" and Opium.............. 43
" Hydrate.... ........69–128–129
" Liniment... ............ 153
" Ointment................ 153
" Eruption from............212
" with Cod Liver Oil........ 69
" and Camphor............ 322
Chloroform..... ............... 154
" Test for............... 10
" Narcosis................ 153
" Gargle................. 293
Chilblains....................... 11
Cough........... .............11–300
" Syrup.... .................. 70
" Oxalate of Cerium in....... 132
" Mixture..........153–189–190–212
Cod-Liver Oil, Digestion of....... 11
Cod-Liver Oil with Hypophosphites and Whiskey............ 44
Cod-Liver Oil with Chloral........ 69
" " " to Cover Taste of.. 159
Counter-irritant................12–128
Ciliary Neuralgia............... 13
Carious Tooth, Death from....... 14
Chorea.......................... 40
Cold Cream...................... 69
Castor Oil Mixture.............. 70
" " Administration of..... 293
" " Emulsion............ 323
Corns........................... 74
Creosote Wine................... 94
Constipation .................... 96
Constipation, habitual.........214-240
Cystitis ......................96–184
" in female........... 215
" in male............ 239
Cardiac Affections............... 99
" Stimulent ............... 157
" Dropsy .................. 185
" Palpitation .............. 213
Caustic Pencils................102-293
Cement for Bottles.............. 102
Cancer.......................... 124
" of Cervix.................. 213
Conderango...................... 126
Catarrh ......................298–238
" of Bladder................ 127
Colds ........................... 187
" in head .................. 153
Cactus ......................... 154
Croup .......................... 160
" and Diphtheria............ 156
Caffein.......................... 157
Chlorate of Potash Mixture....... 190
Conical Cervix................... 210
Carbuncle of Urethra ............ 211
Colic, Infantile.................. 215
Cotton Leaf Tea.................. 262
Catgut Ligature.................. 263
Chorea.......................... 263
Chest Diseases................265–296
Cutaneous Affections............ 265
Copaiba Mixture..............292–293

### INDEX.

PAGE
Chnia Turpentine............293-321
Cedron ........................ 294
Caffeine, Citrate of.............. 295
Coryza, Acute.................... 240
Diarrhœa 12-129-154-186-212-215-241-264.
Diarrhœa, in Tuberculous Patients 242
Diarrhœa Mixture ............... 71
Dysentery.... ......40-97-151-124-129
Diabetes.............294-238-264-297
" Insipidus.................. 42
" Wine in, Test for.......... 217
Dialized Iron, Hypodemically.... 42
Delerium Tremens............... 44
Discharges from the Ear.......... 72
Damiana......................... 157
Dropsy, Cardiac.................. 185
Diphtheria, 19-126-129-157-188-216-241-263-267-292-322-324.
Diptheritc Poison................ 211
Death, Signs of.................. 293
Dubosia and Atropia ............. 293
Dyspnœa......................... 295
Dog Fennel...................... 240
Digestive Derangements.......... 322
Eucalyptus ....................10-128
Ear, Insects in.................. 12
" Discharges from............ 72
" Diseases from Bathing ...... 74
Ether........................... 154
" Danger of................ 13
" Jelly..................... 124
Epilepsy............14-94-95-184-185
Ergotine, hypodermically......... 20
" Suppositories............ 43
" in Uterine Fibroids ..... 44
Electuarium Sennæ Compositum. 41
Epistaxis ........................ 45
Eczema........95-100-124-292-294-239
" of Head .................. 157
Expectorant ..................... 124
Elastic Crayons, of Nit Silver..125-184
Erysipelas ....................127-154
Epithelioma..................... 186
Eruption from Chloral............ 212
Erectile Tumor.................. 216
Foreign Bodies in Throat......... 11
" " " Stomach....... 14
Flatulent Dyspepsia.............. 15
Fuller's Tamarind Electuary..... 41
Fever, Typhoid..............12-45-99
" Intermittent..............94-242
" Malarial................... 125
" Puerperal................. 213
" " Malarial........... 266
" Hay........................ 296
Freckles......................69-211
Facial Neuralgia.............124-266
Febrifuge....................... 125
Fothergill's Cough Mixture....... 153
Funis, Treatment of.............. 212
Fissured Nipples............210-264
Fungus, Vegitation of endometrium 213
Filling for Teeth................ 323
Gonorrhœa ...........11-263-294-324
Glycosuria ...................... 15
Gargle .......................69-243
Gravel ......................... 127
Gout.....................69-124-211
Goitre .......................183-245
Goulard's Cerate................. 210
Gathered Breasts................ 211
Glaucoma....................240-241
Grindelia Squarrosa.............. 240
Hypodermic use of Ether in labor. 16
" " " Ergotine...... 184
" " " Ergotine in metorrhagia ................... 20
Hypodermic use of Silerotinic acid 40
" " " Dyalized Iron. 42
" " " Mercury ...... 160
" Injection, danger of.... 73
" Solution of Morphia and Atrophia......... ...... 95
Hypodermic Syringe Case, Upde-Graff's ....................... 267
Hypodermic use of Magendie's Solution ....................... 262
Hair, growth after death ......... 11
" Tonic..................... 69
" to clense.................. 98
Hæmorrhoids ......40-41-183-262-321
Hoarseness ...................42-210
PAGE
Hepatic Disease.................. 94
Hiccough....................96-183
Hæmoptysis...................... 96
Hydrate of Chloral...........129-264
Hydrophobia...............10-128-262
Hamilton's Tonic................. 185
Hæmotoma of Douglas Pouch ... 211
Habitual Constipation........... 214
Hydrobromic Ether............ 267
Hay Fever....................... 296
Hospital Formulæ............300-322
Hemorrhage at Umbilicus........ 244
Iodized Cod-Liver Oil........... 10
Iodoform, Deodorized............ 40
Iodide Potassium................ 321
Incontinence of Urine........... 41
Ivy Poisoning.................... 94
Itch............................ 323
Intestinal Obstruction........... 95
Ipicac, Substitute for........... 133
Intraliptic Medication........... 184
Ice-cream and Beef Juice........ 213
Infantile Colic.................. 215
Insomnia........................ 216
Iron, Exhibition of............. 296
" Precaution in Administration of....................... 239
Intermittent Fever............94-242
Jamacia Dodwood................ 321
Koumiss......................... 187
Koso ........................... 295
Kidney and Bladder Ailments.... 297
Kameela......................... 324
Labor, Ether in ................. 16
Lister's Antiseptic Medicaments, Formulæ for................. 18
Laboratory Vintage.............. 40
Liver, Abscess of................ 41
Laxative Mixture, Boson's........ 42
Liniment.....................72-190
Laryngitis....................... 294
Local Anæsthesia................ 295
Leeches, to Make Bite........... 239
Migraine........................ 11
Madness ........................ 15
Mineral Waters, home-made...... 16
Metorrhagia ..................... 20
Mercury, elimination of.......... 45
" Hypodermically........ 160
Mother's Marks,.................. 71
Milk and Quinine................. 94
" salt in, for Children....... 98
Malarial Fever.................. 125
" Disorders ..............128-130
Moles .......................... 262
Magendie's Solution.............. 262
Medicated Pencils............... 301
Mammary Abscess, prevention of. 239
Nausea......................10-238
" morphia.................. 154
Nipples, lotion for............... 41
" Sore .......210-262-264-238
Navæ........................70-183
Nervous Sedative................ 94
" Prostration ............ 213
" Exhaustion............. 266
Nervine......................... 126
Neuralgia................241-242-155
" Facial.................. 266
" Supraorbital............ 244
Nitrate of Amyl..............214-126
Nitrate of Silver, Ointment of.... 133
Night Sweats.................... 263
" " in Phthisis......... 268
Nitro-Glycerine.................. 322
Orchitis......................... 14
Ozæna........................... 20
Oil Bath......................... 46
Opium Habit................95-292-297
" Poisoning................. 95
" and Belladonna........... 128
" Nauseous Effects of........ 129
Oxures.......................... 125
Oil of Amber.................... 126
Œsophagus, Clearing of.......... 130
Ophthalmia, Acute .............. 132
Ointment for Piles .............. 153
" in use in N. Y. Hospitals 190
Ovariotomy...................... 214
Ovaries, Prolapse of.............. 215
Ovarian Cyst..................... 215
Oleate of Lead................... 292
Ovarian Dismenorrhœa........... 238
PAGE
Obesity......................... 240
Onions in Consumption.......... 323
Prickley Heat.................... 12
Puerperal Convulsions .........15-262
" Fever .................. 213
" Malarial Fever........... 266
Pimply Face .................... 20
Pepsine from Ostrich............. 40
Poison, to Neutralize............ 42
" Oak.................... 44
" " eruption .......... 70
Poison from Strychnia .......... 97
" " Carbolic Acid........ 322
Pleurisy, Chronic................ 43
Pleuro.Pneumonia ............... 154
Pneumonia....................... 183
Pneumonia, Ergot in............. 72
" Pilocarpine in.......... 154
Poultices........................ 97
" of Cinchona in Camphor 183
Puritus.......................... 124
Phthisis......................... 124
Piles............................ 153
Pepsine, Liquid................. 155
Phenylated Collodium............ 183
Psoriasis........................ 212
Perimetirtes..................... 214
Prolapse of Overies............. 215
Podophyllin..................... 263
Pulsatilla...................264-299
Prurigo.....................40-296
Pencils, Medicated............... 301
Purgatives, saline .............. 238
Prostatic Enlargement........... 238
Pregnancy, nausea in............ 238
Quinine, to lessen quantity....... 13
Quinetum........................ 46
Quinia and Capsicum.........75-156
" " Iron Tonic.......... 238
" for Children........... 242
Rheumatism, Acute Articular 40-46-71-97-101-161-188-292-245.
Rectal Alimination............72-321
Reptile Poisons.................. 213
Rectum, fissure of............... 212
Ringworm ....................... 265
Rosacea, of Face................. 243
Sweating..................14-185
Scrofulous Diseases.............. 17
Sedative Pills ................... 41
Suppositories of Ergotine ....... 43
" " Chloral........ 155
Salicylic Acid.................. 70
" " Mixture............ 69
" " in Rheumatism...... 97
" " Vehicle for......... 322
Soporific........................ 70
Strangulated Hernia............. 70
Sea-Sickness...............71-96-239
Strychnia Poisoning.............. 94
" Antidote..............97-292
" as an Antiemetic......... 125
Sciatica..................94-154-186
Styptic, Pancoast's.............. 95
Sinuses......................... 126
Skin Grafting................... 129
" Diseases................... 298
Stings.......................... 131
Sugar in Urine, Test for.......... 132
Sore Nipples.................123 321
Sick Headache...............158-295
Shock........................... 184
Sim's Speculum, Always at Hand 265
Sneezing....................266-296
Syphilis........................ 299
Sores, painful................... 239
Scabies ......................... 240
Sweating Feet................... 324
Total Abstinance................. 11
Tobacco, death from ............ 11
" Sickness................... 94
Typhoid Fever ...............12-99
" " propagated........ 209
Toe-nail Ingrowing.............. 45
Throat, sore..................... 96
Tape Worm...........99-127-153-297
Thymol ......................... 126
Tetanus....................155-292
Trousseau's Poultice for Rhematic Arthritis ...................... 188
Transfusion......................
Tar, internal use of .............. 293
[illegible]ansy, Oil of..................... 294
[illegible]emperature in Diarrhœa........ 295

PAGE
Tonga .... 300
Trichinæ.... 241
The Telephone.... 259
Toothache.... 321
Uterine Fibroids.... 44
Urethral Fever.... 130
Urea in Blood.... 131
Urine, Glucose in.... 159
Uticaria....240-246-262
Urine, Bile in.... 264
Ulcers of Cervix.... 297
Vaginitis, Membranous, 97-211-243-293
Villates Mixture.... 126
Vomiting in Pregnancy....153-292
" Spirt of Walnut for.... 159
Visceral Disorders.... 240
Whooping Cough, 41-71-95-97-132-183
185-210-212-216-217-239-243-262
Wakefulness.... 71
Wounds, dressing for.... 100
Welles, M. Soelberg.... 238
Yellow Fever.... 130
Zinc, Phosphate of.... 128

**Correspondence.**

Letters, Queries and Replies.—Wise and Otherwise..21-22-48-49-76-77 103-104-134-135-162-163-191-192-193 218-271-272-273-304-326.

**Household Hints and Receipes.**

Artificial flower Barometer.... 51
Butter, artificial to detect.... 50
Blacking, liquid.... 105
" for Boots and Shoes..219-269
Beds, Hygeine of.... 106
" to Keep Bugs from.... 165
Beef Tea, to Make.... 220
Bread.... 302
Bricks, to Blacken.... 313
Cement for Porcelain.... 23
" " Rubber....23-106-303
" " Bottles.... 102
" " Glass.... 105
" " Aquaria Tanks.... 106
" " Uniting Metal to Glass 219
" " Turners.... 270
" " Kerosine Oil Lamps.. 270
" Japanese.... 219
" for Closing Joints in Iron Pipes.... 270
Cough Syrup.... 106
Canine Dystemper.... 107
Carpets, to Restore Color of.... 136
Coloring Edges of Books.... 164
Corn Bread.... 164
Celluloid Collars, to Clean.... 269
Corns.... 269
Chicken Cholera.... 302
Distemper, cure for.... 23
Drying Oil.... 79
Diet.... 105
Damp Rooms.... 107
Engravings, to clean.... 220
Flowers, to keep fresh.... 126
Flowers, to crystalize.... 24
Fruits, to preserve.... 56
" " can.... 78
Fly-glue, to make.... 78
Filtering process.... 79
Furniture Polish.... 136
" Oak.... 302
Glue, Liquid....24-107-164-269-303
" for Damp Places.... 51
" Prepared.... 136
" to Prevent Cracking.... 303
Ground Glass, to Imitate.... 79
Gaslight, on the Eye.... 219
Hands, Wash for.... 24
Hair Tonic.... 50
" Curling Liquid.... 105
Hygiene of Beds.... 106
How to Medicate a Pig.... 270
Honey, to Purify.... 270
Ink, Black....23-51-106-302-303
' Red....23-105-302-303
" Bright Scarlet.... 105
" Green....51-302-303
" Blue....302-3
Ink, Yellow....302-3
" Gold....302-3
" Silver....302-3
" White....302-3
" Indelible....106-303
" to remove....78-219-302
" Invisible, for postal cards.... 79
Iron, to Silver.... 23
" that will not rust.... 136
" to protect from rust....219-269
Incombustible Fabrics.... 303
Iridescent Glass.... 136
Impressions from prints.... 136
Kerosene Liniment....23-107
Lemon Cordial.... 50
" uses of.... 50
Lead Poisoning, to prevent.... 50
Mince Pies.... 23
Milk, Artificial.... 24
" Salt in, for Children.... 50
" Test for.... 50
Mushroom Catsup.... 105
Mouth Wash.... 107
Moths, to Destroy.... 164
Mouldy Bread.... 165
Mucilage, for Postage Stamps.... 269
Oil of Eggs.... 23
Orange Whey.... 78
Pies, Mince.... 23
Poultry, Vermin on.... 24
Paint, to Clean.... 50
Paper, Waterproof.... 78
Painting Iron.... 78
Paint Brushes, to Clean.... 303
Paint, Cheap.... 105
Plants, hot water for.... 79
Piano-keys, to whiten.... 136
Pencils for Glass.... 136
Plating Iron Castings.... 219
Paste.... 269
Press, from clothes wringer.... 302
Polishing Cloth.... 302
Rats, remedy for.... 78
Rancid Butter.... 302
Silvering Iron.... 23
Silver, to polish.... 51
Shampoo fluid.... 78
Stumps, to get rid of.... 78
Skins, to remove wool from.... 105
Starch Gloss.... 105
Stains for wood.... 107
Smoky ceilings to whiten.... 136
Steel Rust.... 136
Sawdust in Art.... 164
Sealing-wax, for Fruit Cans.... 219
Spots, to Remove from Varnish.. 219
Soot Tea, for Roses.... 220
Sweaty Feet, Powder for.... 259
Sorghum, Sugar from.... 270
Tanning Process.... 78
Transparent Paper.... 220
Varnish, for Shoes.... 50
" " Patent Leahter...105-106
" " Gold, for Metals....
Vanillin, Artificial.... 303
Whiskers, to Make Grow.... 50
Waffles....78-164
Waterproof Paper.... 78
Writing and Etching on Glass.... 106
Writing to restore.... 107
White wash.... 136
Water, test for.... 165
Waterproofing boots....269-302
Wash for mouth.... 302
Zincography for Amateurs.... 320

**Editorial.**

Adam.... 221
A Little Politics.... 276
Audiphone, The.... 250
Burns of the Eye.... 82
Children's Legs.... 52
Christmas.... 11
Catch the Moth.... 167
Capital Punishment.... 168
Cremation.... 195
Coroners.... 223
Diphtheria.... 108
Disposal of The Dead.... 138
Don't.... 168
Failing Sight.... 222
PAGE
Free Schools.... 327
Horseback Riding.... 26
Home Treatment of the Eyes.... 52
Injury to the Eyeball.... 25
Indications.... 306
Inhumation.... 249
Medical Advertisements.... 25
Misplaced Sympathy.... 81
Medical Law, the New.... 305
Ovarian Tumors.... 110
Over-Education.... 194
Pedestrianism.... 137
Rain and Rheumatism.... 274
Summering.... 53
Sulphur Bath.... 81
School Buildings.... 137
School Hygien e,I, II....247-274
The Physician.... 166
Yellow Fever.... 80

**Editorial Chit Chat.**

Acorns.... 29
Ausburn Towner....56-277
Attractive.... 83
Always Against the Women.... 112
Aquæ Gloria.... 112
Architecture.... 140
A Nest.... 277
A Mimic.... 278
A Sucker.... 278
Adams, Rev. E. P.... 307
Advertiser, Daily.... 252
Adam Monument.... 331
Bound Bistouries....56-329
Buggy.... 58
Beautiful Place.... 140
Bodines.... 169
Bushels of Cats.... 169
Be Cautious.... 170
Beecher, Thos. K. D. D.... 197
Beecher Supper.... 330
Barbed Fences.... 196
Bernhart, Sara.... 198
Cow Milking Machine.... 57
City Subscription List.... 212
Cooking Club.... 113
Cremation Society....139-169
Cremation at Warren.... 330
Chinese Bill.... 139
Contributions.... 140
Caldwell, Hon. Luther.... 141
Cemeteries and Diphtheria.... 141
Club 101.... 169
Christmas Presents.... 331
Chain Gang.... 197
Camping Number.... 277
Camp Notes.... 281
Cardinal Flowers.... 308
Contributions, The.... 252
Crowded Out.... 329
Dead Heads.... 28
Divorce Threatened.... 56
Dangerous Nuisance.... 83
Dobsons.... 85
Delinquents.... 169
Dixey on Art.... 170
Dwight Inquest.... 170
Dick is Satisfied.... 198
Drum Major.... 277
Desirable Premium.... 307
Designs for the Adam Monument 253
Exposure of Children.... 29
Elmira Prison.... 112
Economy.... 113
Edison.... 278
Eldridge Park.... 251
Fritz.... 278
" Whipping.... 28
" Books.... 56
" On Angels Roosting.... 57
" at School.... 114
" Story.... 139
" Visit to the Conservatory.... 142
" Gets Tired of Being Good.... 252
Flower Planting.... 29
Fourth of July.... 56
Flower Hunting.... 86
Florist's Death—obituary.... 141
Free Love Marriage.... 225
Feat in Balancing.... 279
Great English Physician....30-142
Going Abroad.... 197
Getting Best of an Agent.... 226

| | PAGE |
|---|---|
| Horse, a fine one | 28 |
| " Frighteners | 28 |
| Hospitals and Hard Times | 83 |
| How Many Apples | 112 |
| Home Prescriptions | |
| Household Duties | 278 |
| Harassing | 307 |
| Home for the Aged | 307 |
| Ingersoll | 28 |
| Ithaca | 83 |
| Irregular Consultations | 198 |
| Inscription for Adam Monument | 224 |
| Invention that Failed | 307 |
| In the Woods | 251 |
| Irish Question | 329 |
| Lusus Natura | 83 |
| Life Insurance Companies | 112 |
| Laughing Gas | 113 |
| Life Saving Apparatus | |
| Loan Exhibition | 169 |
| Lawn Mowers | 169 |
| Locomotives and Churches | 277 |
| Locomotive Whistles | 308 |
| Mean Men | 57 |
| Music, Power of | 85 |
| Marriage of Old People | 112 |
| Murray, Rev. W. H. H | 197 |
| Microscope for Sale | 224 |
| Married | 277 |
| Microscopical | 277 |
| " Society | 308 |
| Medical Laws | 309 |
| New Steamer | 83 |
| New Type | 329 |
| Not Exempt | 112 |
| New Cover | 224 |
| Novel Idea | 307 |
| Nicholas W. H. | 251 |
| Not Half | 251 |
| Our Schools | 56 |
| " Vacation | 56 |
| " Boys and Girls | 56 |
| Order asked for | 307 |
| Protective Tariff | 28 |
| Park Church Choir | 29-112 |
| Paroules | 29 |
| Phonograph | 30 |
| Paragraphers | 56 |
| Potter, Wm. C. | 57 |
| Perplexed Coroners | 83 |
| Premiums | 112 |
| Plucky Little Woman | 113 |
| Postage Stamps | 139 |
| Park | 140 |
| Politeness | 170 |
| Penny Nuisance at School | 224 |
| Pin Cushion | 277 |
| Quiet Seat | 279 |
| Reuben | 30 |
| Responses from Horseback Riders | 56 |
| Relatives of Distinguished Men | 112 |
| Reinhold, Dr. H. E. | 139 |
| Rochester Opticians | 330 |
| Spring | 30 |
| Sara Bernhardt | 329 |
| Seeds | 56 |
| Sculpture | 83 |
| State Fair | 84 |
| Subscription Blanks | 112 |
| Stewart's Body | 114 |
| Spring Flowers | 139 |
| Sunday Newspapers | 170 |
| Skating Rink | 331 |
| Sticking Plaster | 197 |
| "Shorty" | 167 |
| Salad Contest | 225 |
| School Buildings | 277 |
| Stop It | 252 |
| Satisfactory Settlement | 329 |
| Trichinæ | 28 |
| Title Page | 28 |
| The Ever-Glorious | 58 |
| That Tent | 83 |
| The Races: | 84 |
| That County History | 113 |
| The Prison Camp | 113 |
| Telegraph, District | 140 |
| To the Profession | 141 |
| The Camping Book | 169 |
| That Book | 197 |
| That Monument | 197 |
| Telegraph Boys | 224 |
| The Fourth | 278 |
| The City | 279 |
| The Klan | 329 |
| Those Two Societies | 307 |
| That Home | 252 |
| Unconscious Hilarity | 169 |
| Vacation in June | 28 |
| Valorous, but in discreet | 278 |
| Volume xvi | 330 |
| Which? | 56 |
| Woodchuck | 57 |
| Well Answered | 83 |
| Wliiams, John D | 139 |
| Williams, A. L. (obituary) | 309 |
| What's in a Name? | 279 |

### SUBSCRIPTIONS RECEIVED

To April 1, 1878.

In this column we will credit every subscription received during the last quarter. Subscribers will please notify us immediately if their payments are not duly credited. The State only is given—this, with the full name of the party subscribing, will be sufficient to indicate who is meant:

NEW YORK—Dr W F Cooper, Bundy Bro's, Jesse Owen, A Houghton Jr, W O Osborne, Dr S G Ellis, Wm Nattall, Walter Booth, H B Berry, S H Ainsworth, Mrs H S Halstead, J Q Adams M D, S M Prentice, Dr J H Banker, Jesse L Cooley, Dr N R Seeley, H R Rogers, Dr G W Little, Dr Van Horne, O C Clark, Dr R L Van Kleek, Ira D Hopkins, M Van Buren, F M Pasco, Hattie TenBroeck, Dr T A Wales, Dr W Gulick, J M Shoemaker, E D Mills, Dr Wm Coryell, L E Goulding, H G Eastman, Dr S X Cotrell, Dr S M Eddy, Dr S A Hibbard, H L Bacon, Dr J C Dixon, Dr D Devening, Dr S Streets, S M Fassett, Dr J M Hagey, L E Hewett, Dr B F Beardsley, C T Thro, Dr D Everett, A B Chamberlin, Dr C H Wetmore, Fred K Jackle, Mary A Potter, Mrs M L Keeney, J D F Slee, Stuart & Beach, W E Hart, T W Elmore, Chas H Gridley, A Romer, Howard Baker, Mrs J C Flett, W F Wentz, S DeWitt, Geo Dorn, W A Kingsbury, N P Fassett, M S Palmer, G P Rawson, J H Barney, Mrs M S Hagadorn, F G Hall, Dr F Darby, E B Youmans, Thomas Burns, Thomas Perry, Chas Rapelyea, S S Hamlin, W M Sanders, Ingraham Bros., J M Robinson & Sons, J L McDowell, A Diven, Dr Parkhurst, Seymour Dexter, Dr Leonard, A R Bunn, Newton Graves, Mrs R Seeley, Mrs Polly Caldwell, Mrs J F Dayton, Dr J W Thompson, Mrs L Lowman, Mrs J M Preston, Mrs T W Crane.

PENNSYLVANIA—Mrs J Eastermarks, D P Miller, J B Chandler, R F Potter, J R Shellenberger, J H Shearer, Alex Beede, W C Hyde, S R Stibgen, R J Wisner, Dr D J Hillbish, I X Grier, Dr J H Wehner, Dr I N Kerlin, A J Semmes, C K Dickinson, Jas Furguson, Dr J Stickel, Dr Jno Wilson, Silas X Billings, W H Nicholas, W C Richards, H G Smyth, T W Sampson, C S Sellard, Robert Thomas, Henry C Ayer, Dr J F Kocher, A A Root, S Griffin, A S Low, Reinhart Smith, Dr C E Sayles, Dr L M Willard, Dr J W Figart, K A Lovell.

ALABAMA—Dr J R Holley, J O Ziegler, W K Chambers.

ARKANSAS—Drs Hooper & Breysacher.

CALIFORNIA—Dr J F Gibbon, Dr G L Simmons.

CONNECTICUT—S A Hubbard.

FLORIDA—Dr N C Stevens, C L Robinson, Dr A J Wakefield, E T Sabal, Calvin Oak, Dr R D Lewis.

ILLINOIS—Jno R Millar.

INDIANA—Jno Drury.

IOWA—Dr J Butts, Thos Up de Graff.

KANSAS—Dr G D Maxson, D G Griswold.

KENTUCKY—J L Newberry.

MASSACHUSETTS—Alfred Wood.

MICHIGAN—A Ganvood, Dr J W Babbitt, Dr Harvey Loomis, Dr L W Fasquelle, Dr DeRoy Dibble, Samuel DuBois, W. S. Livermore, Mrs C J Davis.

MISSOURI—Francis McCabe, Ben H Wilson.

MINNESOTA—Mrs R P Wells.

NEVADA—Dr P T Kirby.

NEW HAMPSHIRE.—Dr S M Emery.

NEW JERSEY—Jno M Atwater, Jas F Norwood, Garrett Broadhead.

NORTH CAROLINA—S Fountain,

OHIO—Q Bradley, Dr Jno Senseman, Dr W M Chesney, Jno A Novinger, Dr F S Hillbish.

SOUTH CAROLINA—D L Anderson, C G Finley.

TENNESSEE—Dr P D Simms, Dr A P Grimstead, I B Walton.

TEXAS—W S Robinson, F L Dickerson.

WISCONSIN—F A Canfield, L M Grover.

WASHINGTON—Surgeon General's Office.

CANADA—Jno L Davis.

